# Castleman Disease: A Rare Lymphoproliferative Disorder With Diverse Clinical Presentation, Diagnosis, and Treatment Approach

**DOI:** 10.7759/cureus.69149

**Published:** 2024-09-11

**Authors:** Jay P Patel, Deep P Patel, Trishul H Amin, Rushikesh K Dave, Daksh Hardaswani, Faizanali Saiyed, Rushita J Goswami

**Affiliations:** 1 Research, Chirayu Medical College and Hospital, Bhopal, IND; 2 Research, AMA School of Medicine, Makati, PHL; 3 Radiology, Sardar Vallabhbhai Patel Institute of Medical Sciences and Research (SVPIMSR), Ahmedabad, IND; 4 Research, Jawaharlal Nehru Medical College, Wardha, IND; 5 Research, Odessa National Medical University, Odessa, UKR; 6 Geriatrics, Bhavsinhji District Hospital, Porbandar, IND

**Keywords:** castleman disease pathophysiology, castleman variant of poems syndrome, diagnosis of castleman disease, imcd diagnostic criteria, multicentric castleman disease (mcd), sites of castleman disease, tafro syndrome, unicentric castleman disease

## Abstract

Castleman disease (CD) includes rare and intricate lymphoproliferative disorders characterized by the abnormal growth of lymph nodes and immune system disturbances. It primarily presents in two forms: unicentric Castleman disease (UCD), which affects a single lymph node area, and multicentric Castleman disease (MCD), which involves multiple lymph nodes and systemic manifestations. The disease's underlying mechanisms are often linked to immune system irregularities, especially involving interleukin-6 (IL-6). The condition was first documented by Dr. Benjamin Castleman in 1954, laying the groundwork for understanding this complex disorder.

MCD can be further divided into idiopathic MCD (iMCD), which includes thrombocytopenia, ascites, fibrosis, renal impairment, and organ enlargement (TAFRO) syndrome, and human herpesvirus-8 (HHV-8)-associated MCD, which can occur in individuals with or without HIV. The prevalence of CD shows a higher occurrence of UCD, with the disease typically presenting in individuals in their fifth to seventh decades of life and being more common in areas with high HIV prevalence. The clinical presentation of CD can include symptoms such as swollen lymph nodes, fever, anemia, and systemic inflammation. Diagnostic challenges arise due to the disease's rarity, and its symptoms overlap with other conditions. Treatment approaches differ based on the subtype. UCD generally responds favorably to the surgical removal of the affected lymph nodes, while MCD often requires antiviral treatments, interleukin-6 (IL-6) inhibitors, and new biologic therapies. Recent advances in treatment, including innovative biologic agents and combination therapies, offer promising prospects for improving patient outcomes. Accurate diagnosis and customized treatment strategies are essential for the effective management of this complex disease.

## Introduction and background

Castleman disease (CD) is a rare and complicated category of lymphoproliferative illnesses marked by aberrant lymph node development and immune system dysfunction [1]. It takes two forms: unicentric, which affects a single lymph node area, and multicentric, which involves numerous lymph nodes and frequently causes systemic symptoms. The disease's underlying processes are unknown; however, it is linked to an abnormal immunological response, specifically interleukin-6 (IL-6) [1]. It was first described by Dr. Benjamin Castleman in 1954, who identified it as a distinct lymphoproliferative disorder characterized by enlarged lymph nodes with unique histopathological features. His initial reports laid the foundation for understanding this rare and complex condition [1]. Castleman disease is categorized into two main types: unicentric and multicentric [2]. Multicentric Castleman disease (MCD) is further divided into three subtypes: idiopathic, which includes thrombocytopenia, ascites, fibrosis, renal impairment, and organ enlargement (TAFRO), not otherwise specified (NOS), and human herpesvirus-8 (HHV-8)-associated, which can occur in HIV-positive or HIV-negative groups [2].

The epidemiology of CD is not well understood, with males being somewhat more impacted by multicentric Castleman disease (MCD) than females, although unicentric Castleman disease (UCD) has no gender predilection [1]. The estimated yearly incidence of UCD and MCD in the United States is between 4,300 and 5,200 cases; however, some research implies a lower frequency [1]. In the United States, approximately 23% of Castleman disease cases are estimated to be multicentric, translating to about 1,001-1,192 instances, and 77% are presumed to have unicentric disease. Although the confidence limits are wide due to various assumptions, these estimates align with other methodologies [3]. In the United States, the incidence has been estimated at five per million patient-years, with instances occurring in persons of all ages and the median age of onset being in the fifth to seventh decades [3]. It insinuates that places with a high HIV prevalence, notably sub-Saharan Africa, may have a greater incidence of HHV-8-associated MCD [3]. CD clinical features include lymphadenopathy, fever, night sweats, fatigue, weight loss, anemia, thrombocytopenia, hypergammaglobulinemia, ascites, hepatosplenomegaly, skin rash, hyperpigmentation, peripheral edema, polyneuropathy, pericardial effusion, hypertension, renal dysfunction, and proteinuria. Polyneuropathy, organomegaly, endocrinopathy, myeloma protein, and skin changes (POEMS), TAFRO, HIV, HHV-8, autoimmune illness (such as systemic lupus erythematosus and rheumatoid arthritis), and cancer are all related to CD and malignancy, particularly among immunocompromised individuals [4].

The majority of research reports that the unicentric type is the most likely neoplastic. The most common cytokine associated with CD for systemic symptoms is interleukin-6 (IL-6) [5]. Researchers have identified that in HHV-8-associated MCD, the upregulation of nuclear factor kappa B (NF-κB) by viral proteins such as viral Fas-associating protein with death domain-like interleukin-1-converting enzyme (v-FLICE) and viral microRNA-K1, along with the upregulation of vascular endothelial growth factor (VEGF) and other factors by a viral G-protein-coupled receptor, may contribute to the disease's pathogenesis. These processes promote B-cell and plasma cell proliferation and angiogenesis and trigger an acute-phase reaction [5]. Kaposi sarcoma herpesvirus (KSHV) infection is specifically associated with immunoglobulin (Ig) M lambda (λ)-expressing B cells, where it can upregulate variable-diversity-joining (V(D)J) recombination and shift Ig kappa (κ) expression to Igλ in B lymphocytes. This virus encodes numerous genes that modulate cell pathways, promoting cell growth, proliferation, and survival, which are key factors in the development of KSHV-MCD, and has high plasma levels of human IL-6 (hIL-6), IL-10, viral IL-6 (vIL-6), and serum C-reactive protein (CRP) [5]. The detailed pathogenesis is discussed below in the discussion section. Diagnosing Castleman disease remains challenging due to its rarity and diverse clinical presentations, often leading to delays in recognition and treatment. Current gaps include a lack of standardized diagnostic criteria and a limited understanding of the disease's underlying mechanisms, highlighting the need for further research.

## Review

Method

The review method involved a comprehensive search of PubMed and Google Scholar databases for articles published from 2013 to the present date. Relevant studies on Castleman disease were selected based on their focus on clinical features, pathogenesis, diagnostic challenges, and treatment approaches. I have screened and collected the articles for review with the assistance of just one other person. This review thoroughly covers current advances in the knowledge, diagnosis, and therapy of Castleman disease.

Castleman disease (CD) encompasses various lymphoproliferative disorders with distinct clinicopathologic features [5]. The unicentric type is characterized by hyaline-vascular (HV) and plasma cell histology, with microscopic changes such as atretic follicles with hyalinization and lymphodepletion, concentric "onionskin" appearance of circumferential mantle zone cells, penetrating vessels with a "lollipop" appearance, and increased vasculature proliferation [5]. The multicentric type is characterized by hypervascular/plasmacytic variant histology. The HHV-8 type is rich in plasma cell, and TAFRO has mixed hypervascular and plasmacytic change [5]. The key cytokine involved is IL-6. The pathogenesis of multicentric Castleman disease (MCD) involves the activation of the JAK-STAT3 and PI3K/Akt/mTOR signaling pathways [6]. A pathway analysis for UCD and idiopathic MCD (iMCD) patients discovered that the genes associated with the mitogen-activated protein kinase (MAPK) pathways (fatty acid synthase {*FAS*}, platelet-derived growth factor receptor B {*PDGFRB*}, fibroblast growth factor receptor 3 {*FGFR3*}, neurofibromatosis type 1 {*NF1*}, and transforming growth factor beta receptor 2 {*TGFBR2*}) were the most often damaged in UCD [7]. In iMCD, genes in the MAPK pathway were also mutated (protein tyrosine phosphatase receptor type R {*PTPRR*}, *ERBB2*, *FAS*, serine/threonine kinase 3 {*STK3*}, and *TGFBR2*) [7].

Caspase recruitment domain-containing protein 11 (*CARD11*), found on chromosome 7p22, is crucial in regulating the NF-κB signaling pathway [8]. Activating mutations in *CARD11* have been observed in B-cell lymphomas and B-cell lymphocytosis, where they drive cellular proliferation [9]. *CARD11* is also involved in autoimmune lymphoproliferative syndrome (ALPS) and ALPS-like syndromes, which can share clinical features with CD. Additionally, recent sequencing studies have identified genetic mutations linked to CD on chromosome 7, including BRAF (MAPK pathway, 7q34), WEE2 (7q34), lysine methyltransferase 2E {*KMT2E*} (chromatin remodeling, 7q22), histone deacetylase 9 {*HDAC9*} (chromatin remodeling, 7p21), and dynein axonemal heavy chain 11 {*DNAH11*} (cell function, 7p15) [9,10]. HHV8-associated MCD is driven by the virus's ability to evade immune detection and trigger a cytokine storm, particularly through the production of vIL-6, which enhances cell proliferation and inflammation. The viral genome also encodes proteins that directly activate signaling pathways such as JAK/STAT and MAPK, which are critical for the survival and expansion of infected cells [10,11]. This dysregulation results in lymphoid hyperplasia, increased immunoglobulin production, and neo-angiogenesis, leading to the characteristic pathological changes in MCD [11]. This process multiplies plasmablasts and is followed by extensive viral replication, cell lysis, and excessive cytokine release, all of which promote cell-to-cell transmission and proliferation. The result of such a vicious loop is an exponential increase in hIL-6 and vIL-6, both locally and systemically. The binding of hIL-6 and vIL-6 to IL-6 receptors activates the JAK/STAT and MAPK pathways at the cellular level [12].

UCD commonly presents with primary lymph nodes located in the neck or abdomen, with many cases discovered incidentally during imaging. Systemic symptoms such as anemia, fatigue, and abdominal discomfort are observed in nearly half of the patients. Diagnostic imaging often reveals homogeneous hypoechoic nodules with rich blood flow [13]. All UCD patients in the study underwent surgical resection, with a favorable outcome, as no relapses or disease progression was reported after a median follow-up of 4.08 years [13]. Hu et al.'s study showed that 23 patients diagnosed with MCD presented with a painless neck mass, multiple lymphadenopathies, and elevated inflammatory cytokines but no systemic symptoms [13].

In the study described by the author, the sex ratio among patients with UCD was balanced, while both MCD cases were male. UCD typically occurs at a younger median age compared to MCD. UCD often presents as the hyaline-vascular (HV) subtype, while MCD usually manifests as plasma cell or mixed subtypes; however, both MCD patients in this study exhibited the HV subtype [13,14]. UCD generally shows a homogenous phenotype with isolated lymph nodes, but 45% of UCD patients in this cohort had systemic symptoms, a higher proportion than typically observed in adults. Growth retardation, potentially linked to chronic inflammation, was noted as a significant clinical feature in one patient [14]. MCD symptoms include lymphadenopathy, anemia, splenomegaly, renal dysfunction, fever, pulmonary illness, pleural effusion/ascites, thrombocytopenia, skin lesions, chronic pancreatitis, diabetes, heart failure, and vascular events. The clinical and histological aspects of IgG4-related illness can be highly similar, and even indistinguishable, from those of MCD [15]. KSHV-MCD is more frequent among HIV-positive patients; however, it can also occur in people who do not have HIV. During inflammatory "flares" in KSHV-MCD, the KSHV viral load in peripheral blood is nearly always high, and KSHV-MCDs are plasma cell types [16,17].

TAFRO syndrome

TAFRO syndrome is a Castleman disease variation with a different set of symptoms, including thrombocytopenia, anasarca (widespread edema), fever, renal failure, and organomegaly. Thrombocytopenia (low platelet count) and anasarca (widespread swelling or edema) are frequently among the first symptoms to emerge. These symptoms often appear early in the illness course and can be useful markers for early diagnosis [18]. Unlike conventional MCD, TAFRO syndrome frequently exhibits more severe clinical characteristics, such as fast disease progression and greater fatality rates. One of the primary distinctions between MCD and TAFRO disease is the cytokine profile. While both illnesses include high levels of interleukin-6 (IL-6), TAFRO syndrome has more dramatic increases in a broader spectrum of cytokines, including vascular endothelial growth factor (VEGF) and C-reactive protein (CRP), which contribute to its aggressive clinical presentation [18]. In contrast, IgG4-related illness is not characterized by a high fever or a significant increase in CRP [19].

POEMS syndrome

POEMS syndrome is a rare, multisystem disorder characterized by polyneuropathy, organomegaly, endocrinopathy, monoclonal gammopathy, and skin changes [20]. It is distinct from UCD, which generally involves a single lymph node, and MCD, which affects multiple lymph node regions. POEMS is marked by a combination of peripheral neuropathy and organomegaly, along with systemic symptoms due to the overproduction of cytokines, especially vascular endothelial growth factor (VEGF) [20]. The elevated VEGF levels in POEMS contribute significantly to its pathogenesis and clinical features, leading to vascular leakage and symptoms such as edema, pleural effusions, and ascites. In contrast to MCD, where interleukin-6 (IL-6) is the primary cytokine, POEMS syndrome's cytokine increase is predominantly VEGF, making it an important target for therapeutic approaches [20].

Challenges in diagnosis

The article by Mathew et al. outlines several diagnostic hurdles for CD, especially in cases of UCD with unusual presentations. A major issue is the nonspecific and varied clinical symptoms, which can closely resemble other diseases, potentially leading to misdiagnosis or delayed identification [21]. Diagnostic imaging may not effectively distinguish Castleman disease from other lymphoproliferative disorders or cancers, complicating the diagnostic process further. While histopathological analysis is crucial for an accurate diagnosis, it can be difficult due to the diverse nature of tissue features. Additionally, the rarity of the disease often means that clinicians may not be aware, which can result in delays in appropriate treatment [21]. González García et al. highlight several challenges in diagnosing idiopathic MCD. A key difficulty arises from the overlap of MCD symptoms with those of autoimmune and autoinflammatory diseases, which can make it difficult to pinpoint the condition [22]. The diverse clinical manifestations of MCD, such as systemic symptoms (fever, weight loss, and lymphadenopathy), can be easily confused with other illnesses, leading to delays in diagnosis. Laboratory tests and imaging often provide nonspecific findings, complicating the diagnostic process. While histological evaluation is crucial for diagnosis, the variability in tissue characteristics of MCD can pose challenges. Additionally, the absence of a singular definitive test for MCD necessitates a thorough diagnostic strategy that includes ruling out other potential conditions [22]. So, the disease's rare nature means that clinicians may not always consider it, contributing to delayed or incorrect diagnoses.

Diagnosis

The article by Pertusa Mataix et al. discusses the diagnostic approach for Castleman disease, emphasizing the importance of a multifaceted evaluation. To diagnose Castleman disease, a combination of imaging studies, such as ultrasound, computed tomography (CT), and magnetic resonance imaging (MRI), is used to assess lymph node involvement and disease extent [23]. Histopathological examination through biopsy remains crucial for definitive diagnosis, as it helps in identifying characteristic features of the disease. Blood tests, including markers of inflammation and specific cytokine levels (IL-6, IL-10, CRP, VEGF, and beta-2 microglobulin), are also conducted to evaluate systemic involvement. Observing these tests helps in distinguishing Castleman disease from other similar conditions and tailoring appropriate treatment strategies [23]. The article by Din et al. highlights the essential role of imaging in diagnosing Castleman disease and its various subtypes. Effective diagnosis usually involves using imaging modalities such as CT and MRI. CT scans often display distinctive features, including uniform enhancement and lymph node enlargement, while MRI offers detailed insights into soft tissue involvement and disease extent [24]. Imaging is crucial for identifying solitary lymph nodes in UCD and detecting multiple affected lymph nodes and systemic involvement in MCD. It also plays a vital role in monitoring treatment efficacy and disease progression by observing changes in lymph node size and the emergence of new lesions. Advanced imaging is key for differentiating Castleman disease from other lymphoproliferative conditions and directing appropriate clinical care [24].

The article by Koa et al. emphasizes the importance of 18F-fluorodeoxyglucose positron emission tomography (18F-FDG PET)/CT scans in diagnosing Castleman disease. This imaging technique provides detailed metabolic insights, revealing glucose uptake patterns that help distinguish Castleman disease from other lymphoproliferative disorders. It is crucial for assessing disease extent, staging, and monitoring treatment response by tracking changes in metabolic activity [25].

IgG4 diseases and Castleman disease differentiation

Chen et al. address the diagnostic difficulties in differentiating IgG4-related disease (IgG4-RD) from Castleman disease due to their similar presentations. Both disorders can exhibit lymphadenopathy and elevated serum IgG4 levels, complicating the distinction. Nonetheless, certain diagnostic markers can help differentiate them [26].

IgG4-RD is marked by significantly high serum IgG4 levels and a predominance of IgG4-positive plasma cells in tissue samples, which are not typically observed in Castleman disease. Conversely, Castleman disease is associated with a more extensive cytokine imbalance, including elevated interleukin-6 (IL-6) and vascular endothelial growth factor (VEGF) [26]. Additionally, IgG4-RD may involve systemic conditions such as autoimmune pancreatitis or sclerosing cholangitis, which are not features of Castleman disease. Effective diagnosis relies on a thorough assessment of clinical presentation, serological findings, and histological characteristics to accurately differentiate between these conditions and determine the appropriate treatment approach [26].

Treatment

The approach to treatment depends on the specific subtype of CD.

UCD

The primary treatment for UCD is the surgical resection of the affected lymph node(s). This method offers a high rate of cure and is considered the gold standard for managing this localized form of the disease [16].

MCD

Treatment for MCD is more complex due to its systemic nature. For HHV-8-associated MCD, antiviral therapies such as antiretrovirals for HIV and antiviral agents targeting HHV-8 are crucial. Rituximab, an anti-cluster of differentiation 20 (CD20) monoclonal antibody, has shown effectiveness in treating MCD, particularly in cases where it is not associated with HHV-8 [16].

Idiopathic MCD (iMCD)

This form may respond to interleukin-6 (IL-6) inhibitors such as siltuximab or tocilizumab. If IL-6 inhibitors are ineffective, alternative treatments such as corticosteroids, chemotherapy, or other immunomodulatory agents might be used [27]. Emerging therapies, including the mTOR inhibitors and JAK inhibitors, are also under investigation.

TAFRO Syndrome

For TAFRO syndrome, treatment often involves a combination of immunosuppressive therapies. Corticosteroids are commonly used, and patients may also receive therapies targeting specific cytokines or immunomodulatory treatments tailored to individual needs [16]. The article by Kapriniotis et al. outlines various biologic agents for treating MCD [28]. Rituximab, an anti-CD20 monoclonal antibody, is particularly effective for MCD not associated with HHV-8. Siltuximab, an inhibitor of IL-6, targets the central inflammatory processes of MCD. Tocilizumab, another IL-6 receptor blocker, is used if siltuximab proves inadequate. Daratumumab, which targets CD38-expressing cells, is less frequently utilized but is being studied in clinical contexts [28].

The article by Lurain et al. outlines the treatment approaches for Kaposi sarcoma herpesvirus (KSHV)-associated MCD. Effective management includes targeted therapies to control the viral infection and alleviate symptoms. Antiviral treatments specifically addressing KSHV are essential for tackling the disease's root cause [29]. Additionally, immunomodulatory drugs, such as IL-6 inhibitors and rituximab, are critical for addressing the disease's inflammatory and immune-related aspects. Treatment typically involves a collaborative approach, with continuous monitoring and adjustment based on the patient's response and disease development [29]. The guidelines presented by van Rhee et al. outline a treatment strategy for UCD. The first-line treatment is typically surgical resection, which often leads to high remission rates and is considered curative. In patients ineligible for surgery or with remaining disease, radiation therapy serves as a viable alternative [30]. If surgery and radiation are not suitable, systemic treatments such as corticosteroids or immunomodulatory drugs may be used. Ongoing monitoring is essential to detect any recurrence or complications. The guidelines stress the importance of tailoring treatment plans to individual patient needs and disease specifics for the best results [30].

Another notable development is the investigation of innovative therapies, such as combination treatments and emerging biologic agents, designed to improve effectiveness and lower relapse rates. Furthermore, progress in personalized medicine is enabling more customized treatment strategies tailored to each patient's unique profile and disease specifics [31]. These advancements mark significant progress in the management of Castleman disease, providing renewed optimism for enhancing patient care. Recent advancements in biologic agents for Castleman disease include several promising options [31]. Daratumumab, a monoclonal antibody targeting CD38, has shown potential in managing the disease by influencing tumor cell proliferation and survival. Ixazomib, a proteasome inhibitor, can be effective when used in combination with other therapies, addressing cellular mechanisms implicated in Castleman disease. Emicizumab, primarily used for hemophilia, is also under investigation for its possible benefits in Castleman disease due to its effects on immune responses. Bruton's tyrosine kinase (BTK) inhibitors are being explored for their ability to modulate immune activity and reduce inflammation. Additionally, chimeric antigen receptor (CAR) T-cell therapies, which involve engineering T cells to target and destroy disease cells, are being studied for their effectiveness in treating CD [31].

Prognosis

The article by Huang et al. examines various factors that impact the prognosis of CD, highlighting critical determinants of patient outcomes [32]. The disease subtype is a major factor, with UCD typically showing a better prognosis due to its localized nature and the potential for complete surgical removal. On the other hand, MCD presents a more challenging prognosis due to its widespread involvement and associated complications [32]. Other influential factors include the presence of comorbid conditions, the response to treatment, and the overall health of the patient. Moreover, cytokine levels such as IL-6 and VEGF are important, as elevated concentrations often reflect more severe MCD and are linked to poorer outcomes. Monitoring these biomarkers is essential for evaluating disease activity and tailoring treatment, which can significantly impact prognosis. Early diagnosis and timely treatment interventions are crucial for improving disease management and survival rates [32]. The prognosis for patients with both HIV and Castleman disease can be significantly influenced by the interaction between the two conditions. HIV-infected individuals often face more severe disease progression, as the virus can intensify inflammatory responses and complicate treatment strategies [33]. Timely diagnosis and thorough treatment are crucial for improving outcomes in these cases. Additionally, managing HIV effectively and closely monitoring disease activity are vital for achieving better long-term results [33].

## Conclusions

In summary, Castleman disease presents a complex array of challenges due to its diverse subtypes and variable clinical presentations. Both unicentric and multicentric forms of the disease require distinct management strategies, with unicentric Castleman disease generally offering a more favorable prognosis due to its localized nature and the effectiveness of surgical intervention. Conversely, multicentric Castleman disease, particularly when associated with HHV-8 or HIV, poses more significant treatment difficulties and generally has a more guarded outlook. Recent advances in diagnostic techniques, such as advanced imaging and biomarker monitoring, have improved disease detection and management. Additionally, emerging therapies and personalized treatment approaches are providing new avenues for improving patient outcomes. Continued research is essential for refining these strategies and enhancing our understanding of Castleman disease to ultimately improve patient care and prognosis.
